# Novel involvement of RhebL1 in sphingosylphosphorylcholine-induced keratin phosphorylation and reorganization: Binding to and activation of AKT1

**DOI:** 10.18632/oncotarget.15364

**Published:** 2017-02-15

**Authors:** Hyun Ji Kim, Hyun Jung Byun, Mi Kyung Park, Eun Ji Kim, Gyeoung Jin Kang, Chang Hoon Lee

**Affiliations:** ^1^ College of Pharmacy, Dongguk University, Seoul 100-715, Republic of Korea

**Keywords:** sphingosylphosphorylcholine, RhebL1, AKT1, keratin reorganization, migration

## Abstract

Sphingosylphosphorylcholine induces keratin phosphorylation and reorganization, and increases viscoelasticity of metastatic cancer cells such as PANC-1 cells. However, the mechanism involved in sphingosylphosphorylcholine-induced keratin phosphorylation and reorganization is largely unknown. Sphingosylphosphorylcholine dose- and time-dependently induces the expression of RhebL1. The involvement of RhebL1 in sphingosylphosphorylcholine-induced events including keratin 8 (K8) phosphorylation, reorganization, migration and invasion was examined. Gene silencing of RhebL1 suppressed the sphingosylphosphorylcholine-induced events and overexpression of RhebL1 enhanced those events even without sphingosylphosphorylcholine treatment. We examined whether the G protein function of RhebL1 induces K8 phosphorylation using constitutively active RhebL1Q64L and dominant negative RhebL1D60K. G protein activity of RhebL1 is involved in sphingosylphosphorylcholine-induced K8 phosphorylation. We found that RhebL1 binds and activates AKT1. G protein activity of RhebL1 is involved in the binding and activation of AKT1. MK2206 (AKT inhibitor) and gene silencing of AKT1 inhibited the sphingosylphosphorylcholine-induced events, whereas overexpression of activated-AKT1 induced K8 phosphorylation, reorganization, migration and invasion even without sphingosylphosphorylcholine treatment.

The collective results indicate that RhebL1 is involved in sphingosylphosphorylcholine-induced events in A549 lung cancer cells via binding to AKT1 leading to activation of it. These results suggest that suppression of RhebL1 or inhibition of RhebL1′s binding to AKT1 might be a novel way that prevents changes in the physical properties of metastatic cancer cells.

## INTRODUCTION

Lung cancer occurs mostly in the second commonest cancer and corresponds to 14% of new cancers [1]. Adenocarcinomas are the most common type of non-small cell lung cancer, which currently accounts for approximately 85% of all lung cancer [2]. Lung cancers generally metastasize to the bone, brain, liver, and adrenal gland [3].

Metastasis accounts for more than 90% of cancer-related death [4]. However, development of therapeutics against metastasis is a challenge due to the incomplete understanding of the process involved. Recently, researchers have focused on the differences between normal cells and cancer cells in an attempt to find compounds that change the physical properties of metastatic cancer cells [5–10]. In an earlier study, we showed that FTY720 reverses the SPC-induced changes of viscoelasticity in PANC-1 cancer cells [11].

SPC is a multifunctional molecule identified in the cardiovascular system, immune system, central nervous system, and skin [12–14].” Increased levels of SPC have been found in atopic dermatitis, Niemann–Pick disease (NPD), and malignant ascites of patients with tumors [15–17]

Changes of viscoelasticity are associated with phosphorylation and reorganization of the keratins in various metastatic cancer cells including A549 cells and PANC-1 cells [5, 18–20]. Shear stress induces reorganization of the keratin network in A549 cancer cells through protein kinase C [21]. Several endogenous or exogenous compounds such as SPC, LTB_4_, or cerulein can induce phosphorylation and reorganization of K8 in epithelial cancer cells [5, 18–20]. The mechanical deformability of cancer cells induced by keratin reorganization facilitates migration and invasion of cancer cells through the limited space in cancer tissues [5, 6, 8].

Phosphorylation of K8 at serine 431 (S431) is important in perinuclear reorganization of K8 in PANC-1 and A549 cancer cells [18]. Protein kinases such as c-Jun N-terminal kinase (JNK) and extracellular signal-regulated kinase (ERK) participate in phosphorylation and reorganization of K8 in cancer cells. ERK and JNK phosphorylate K8 at S431; whereas protein phosphatase 2A (PP2A) reverses phosphorylation of S431, and inactivates ERK and JNK by dephosphorylation [8, 22–24]. However, overall phosphorylation and reorganization of keratin remain unclear.

RhebL1 belongs to the ras superfamily of G proteins and is highly conserved across many species [25]. Mammalian target of rapamycin (mTOR) is activated by the small G protein Rheb1 [25–27]. Once activated, mTOR catalyzes the phosphorylation of several downstream effectors which, in turn, influence growth [28]. Mammals harbor two Rheb genes: Rheb1 and RhebL1 (Rheb2) [29]. The two proteins are 52% identical in amino acid composition and are thought to be redundant in function. However, despite having apparently functionally conserved roles, differences with regard to localization and expression pattern of these genes have been reported [30].

In this report, we describe SPC induction of RhebL1 expression in lung cancer cells. RhebL1 is involved in SPC-induced K8 phosphorylation and reorganization leading to enhanced migration and invasion. RhebL1 binds to and activates AKT1, which is involved in SPC-induced K8 phosphorylation and reorganization.

## RESULTS

### SPC induces RhebL1 expression in lung cancer cell lines

**Figure 1:**
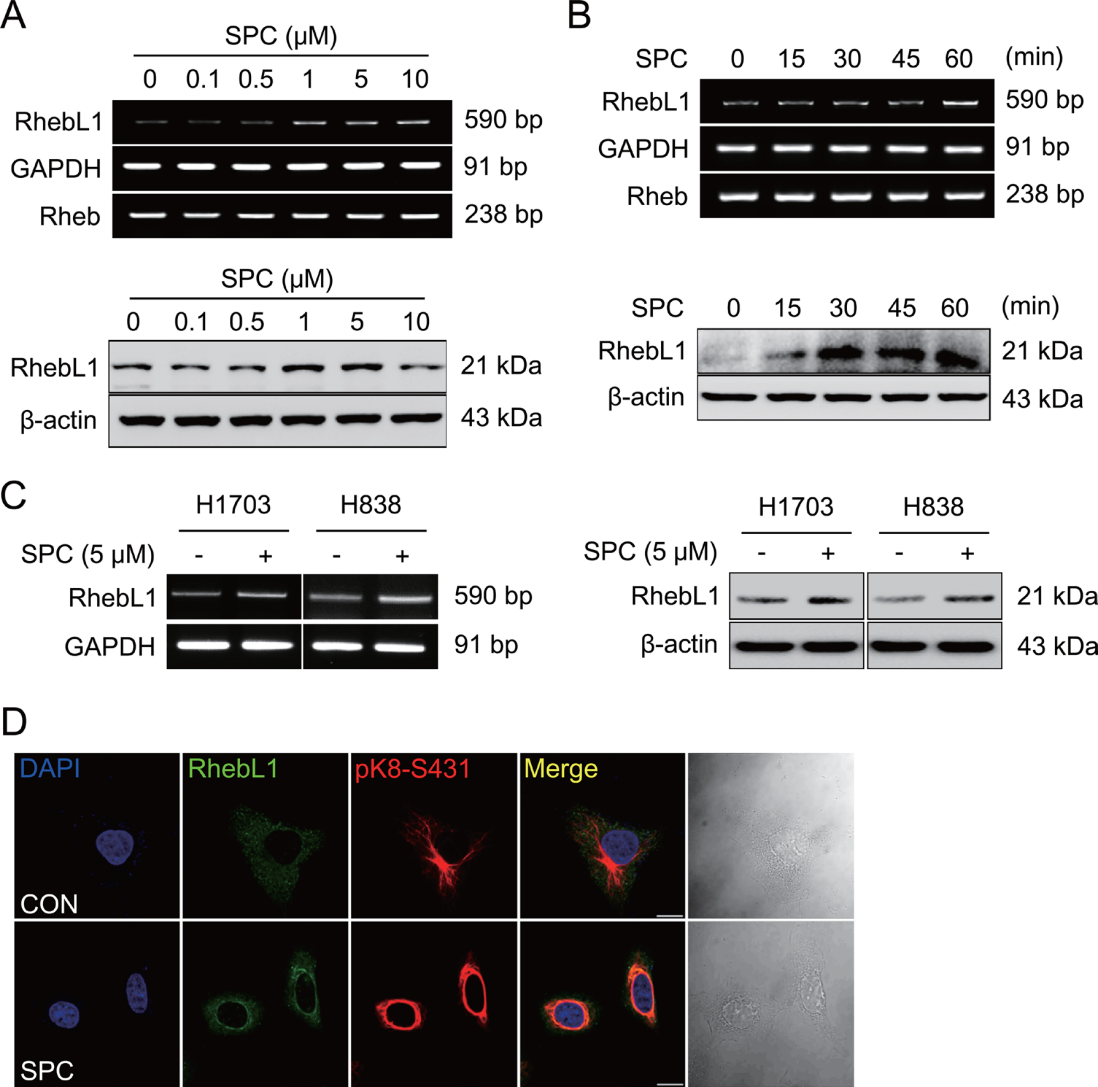
SPC induces RhebL1 expression in A549 cells (**A**) Dose-dependent expression of RhebL1 mRNA and protein in A549 cells stimulated with the indicated concentrations of SPC for 1 h. (**B**) Time-dependent expression of RhebL1 mRNA and protein in A549 cells were treated with 5 μM SPC for the times indicated. (**C**) Effects of SPC on expression of RhebL1 mRNA and protein in H1703 and H838 cells. (**D**) Confocal microscopic analysis of RhebL1 and phosphorylation of K8 S431 in A549 cells stimulated with 5 μM SPC for 1 h. Nuclei were stained with DAPI (blue). Scale bars, 10 μm. In (A)∼(C), gene expression of RhebL1 was detected by RT-PCR and Western blotting analysis.

We found that the expression level of RhebL1 was increased in cells treated with SPC through microarray experiments (data not shown). SPC induced expression of RhebL1 in a dose- and time- dependent manner in A549 cells (Figure 1A and 1B, Supplementary Figure 1A and 1B). The induction of RhebL1 expression was also observed in H1703 and H838 lung cancer cells (Figure 1C, Supplementary Figure 1C). RhebL1 expression was examined by confocal microscopy. A549 cells showed the typical cytoplasmic pattern of keratin filaments and RhebL1 expression was weak (Figure 1D). Interestingly, RhebL1 localization was similar to that of keratin filaments. SPC induced RhebL1 expression and the reorganization of keratin filaments to a perinuclear, ring–like structure (Figure 1D). Interestingly, the expression patterns of RhebL1 induced by SPC were consistent with perinuclear ring formation of K8 in confocal microscopy. These results suggest that RhebL1 might be involved in SPC-induced keratin reorganization.

### RhebL1 is involved in SPC-induced K8 phosphorylation, reorganization, migration and invasion of lung cancer cells

**Figure 2:**
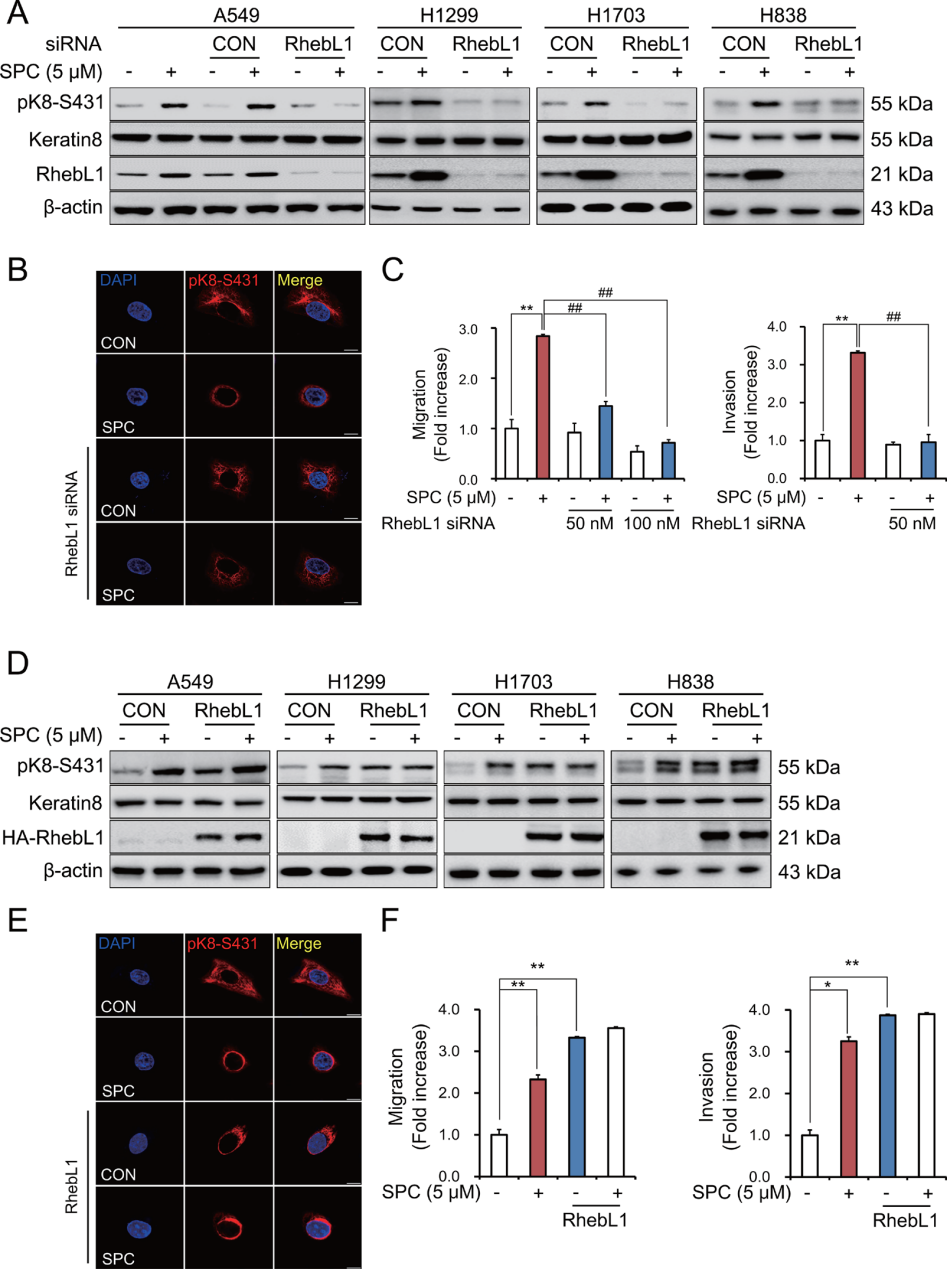
RhebL1 is involved in the SPC-induced K8 phosphorylation and reorganization of A549 cells (**A**) Effect of RhebL1 siRNA on SPC-induced K8 phosphorylation. For gene silencing of RhebL1, A549, H1299, H838 and H1703 cells were transfected with RhebL1 siRNA (50 nM) and control siRNA (50 nM) and subsequently stimulated with or without SPC (5 μM) for 1 h. (**B**) Effect of RhebL1 siRNA on perinuclear keratin reorganization in A549 cells stimulated with SPC. A549 cells were stained with the indicated antibodies. Nuclei were stained using DAPI (blue). Scale bars, 10 μm. (**C)** Effects of RhebL1 siRNA on SPC-induced migration and invasion in A549 cells. After gene silencing of RhebL1 and treatment of SPC, A549 cells (5 × 10^4^ cells per well) were plated the upper chamber of Transwell insert for migration and invasion assay. The results shown are representative of 3 independent experiments with similar results (*n* = 3). (**D**) Effect of RhebL1 overexpression on SPC-induced K8 phosphorylation. For gene overexpression of RhebL1, A549, H1299, H838 and H1703 cells were transfected with the plasmid containing RhebL1 and control empty vector (4 μg) and then treated with or without SPC (5 μM) for 1 h. (**E**) Effect of RhebL1 overexpression on perinuclear keratin reorganization in A549 cells stimulated with SPC. Scale bars, 10 μm. (**F**) Effects of RhebL1 overexpression on SPC-induced migration and invasion in A549 cells. After gene overexpression of RhebL1 and treatment of SPC, A549 cells (5 × 10^4^ cells per well) were plated the upper chamber of Transwell insert for migration and invasion assay. The results shown are representative of 3 independent experiments with similar results (*n* = 3). *P < 0.05, **P < 0.01 compared with the control group. ^#^*P* < 0.05, ^##^*P* < 0.05 compared with the SPC-treated group.

To investigate the involvement of RhebL1 in SPC-induced events including K8 phosphorylation, reorganization, migration, and invasion, we examined the effects of gene silencing and overexpression of RhebL1 on the SPC-induced events in A549 lung cancer cells. Gene silencing of RhebL1 inhibited SPC-induced phosphorylation of S431 in K8 in lung cancer cells including A549, H1299, H1703, and H838 lung cancer cells (Figure 2A, Supplementary Figure 1D) and reorganization of K8 in A549 lung cancer cells (Figure 2B). In addition, SPC-induced migration and invasion were suppressed by gene silencing of RhebL1 in A549 cells (Figure 2C). In contrast, overexpression of RhebL1 induced phosphorylation of K8 in A549, H1299, H1703, and H838 lung cancer cells (Figure 2D) and reorganization of K8 in A549 cells, even without SPC treatment (Figure 2E). Moreover, overexpression of RhebL1 promoted migration and invasion in A549 cells (Figure 2F). These results suggest that RhebL1 is involved in the SPC-induced events.

### Effects of G protein activity of RhebL1 on K8 phosphorylation, reorganization, migration and invasion

**Figure 3:**
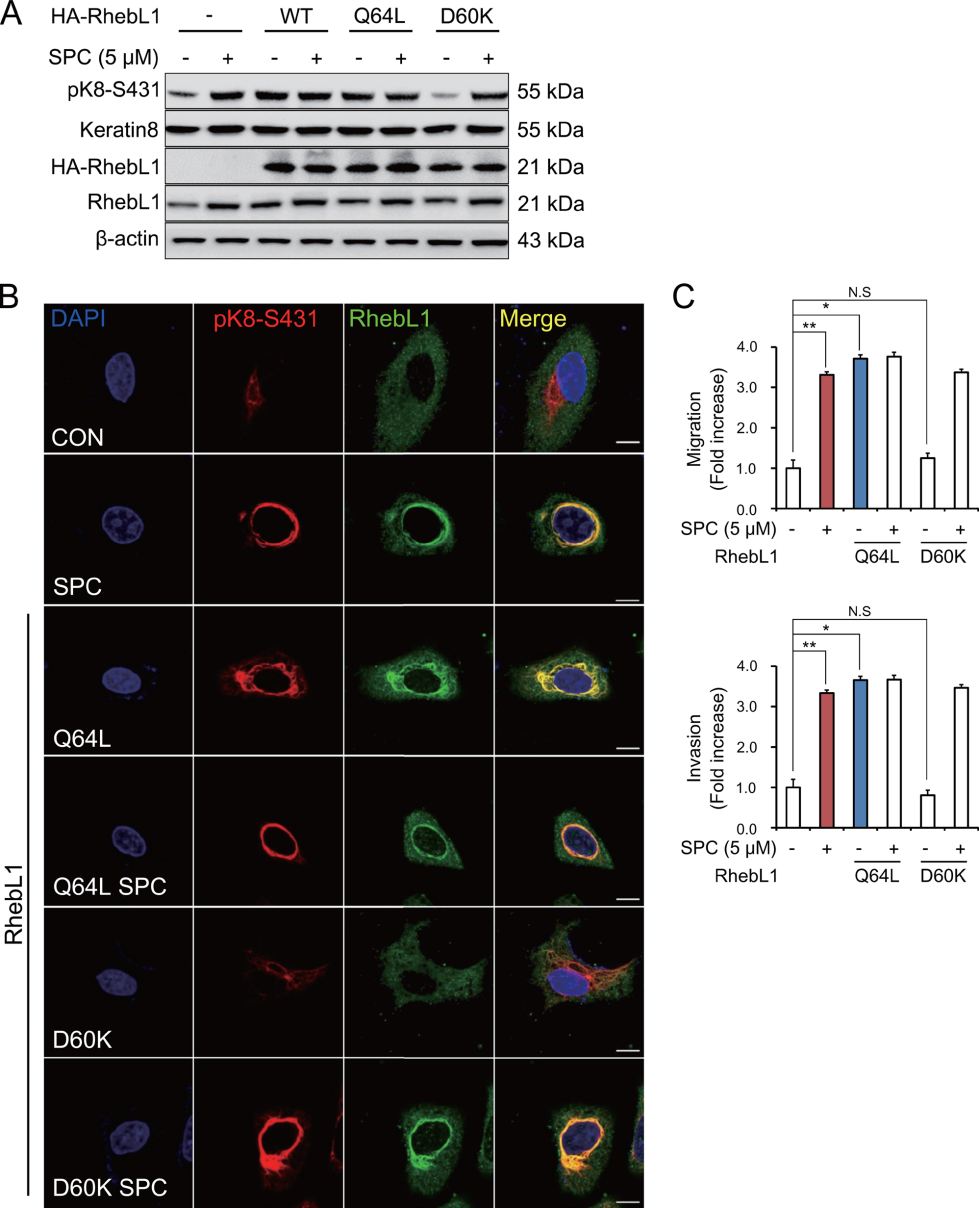
Effects of G protein activity of RhebL1 on K8 phosphorylation and reorganization (**A**) Effect of G protein activity of RhebL1 on SPC-induced K8 phosphorylation. For gene overexpression of RhebL1, RhebL1 D60K (dominant negative) and RhebL1 Q64L (constitutively active), A549 cells were transfected with the plasmid containing RhebL1 and control empty vector (4 μg) and then treated with SPC (5 μM) for 1 h. Cell lysates were analyzed by Western blot. (**B**) Effect of G protein activity of RhebL1 on SPC-induced K8 reorganization. Scale bars, 10 μm. (**C**) Effects of G protein activity of RhebL1 on SPC-induced migration and invasion. The results shown are representative of 3 independent experiments with similar results.(*n* = 3). *P < 0.05, **P < 0.01 compared with the control group. ^#^*P* < 0.05, ^##^*P* < 0.05 compared with the SPC-treated group. N.S corresponds to not significant.

RhebL1 has G protein activity [31]. So, we examined whether G protein activity of RhebL1 could increase SPC-induced K8 phosphorylation. Glutamine (Q) 64 of RhebL1 was site-directed mutated to leucine (Q64L) as the active form in which GTP is constitutively bound and aspartic acid (D) of RhebL1 to lysine (D60K) as the dominant negative form [32] which cannot bind to GTP. K8 phosphorylation was induced by overexpression of RhebL1Q64L but not by D60K without SPC treatment in A549 cells (Figure 3A). K8 reorganization was also induced by overexpression of RhebL1Q64L but not by D60K without SPC treatment in A549 cells (Figure 3B). RhebL1Q64L overexpression enhanced migration and invasion of A549 lung cancer cells without SPC treatment (Figure 3C). These results suggest that G protein activity of RhebL1 is related to SPC-induced K8 phosphorylation and reorganization leading to migration and invasion of A549 cells.

### RhebL1 binds to AKT1 and is involved in AKT phosphorylation

We speculated that RhebL1 might bind to AKT1 directly or indirectly via mTOR since RhebL1 could bind to mTOR and mTOR was phosphorylated by AKT1 [33, 34].

**Figure 4:**
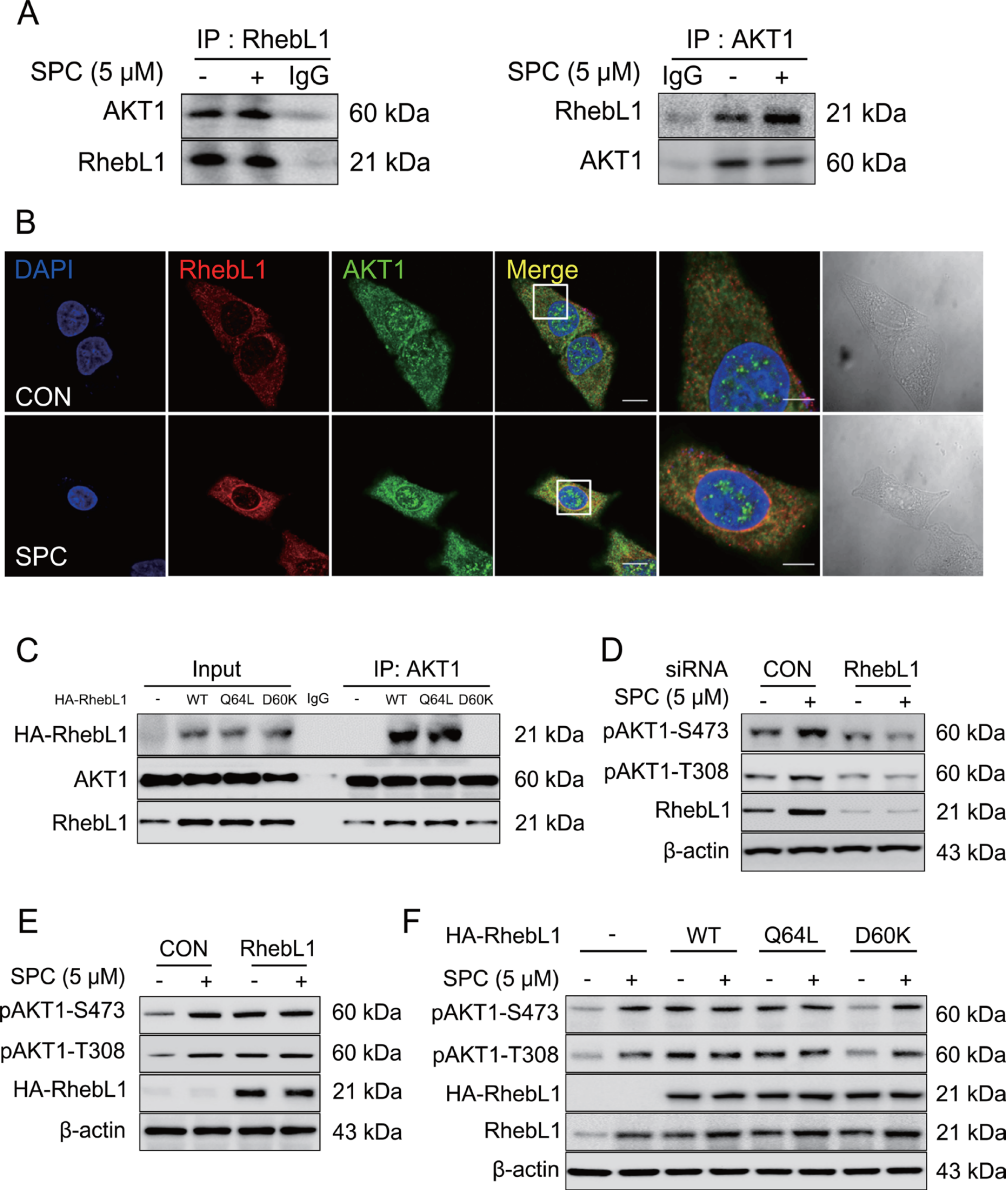
RhebL1 binds to AKT leading to activation of AKT1 (**A**) Binding of AKT1 to RhebL1. Immunoprecipitation with the AKT1 antibody (IP: AKT1) or RhebL1 antibody (IP: RhebL1) were performed on the extracts of A549 cells with or without 5 μM SPC for 1 h. The resulting immunocomplexes were immunoblotted with AKT1 and RhebL1 antibody. (**B**) Confocal microscopic analysis of binding of AKT1 to RhebL1. (**C**) Effect of G protein function of RhebL1 on SPC-induced binding of RhebL1 to AKT1 in A549 cells. Immunoprecipitation with the AKT1 antibody (IP: AKT1) was performed on the extracts of A549 cells transfected with plasmids containing wild type RhebL1, RhebL1D60K or RhebL1Q64L, respectively. The resulting immunocomplexes were immunoblotted with HA and AKT1 antibodies. (**D**) Effects of RhebL1 siRNA on AKT activation. (**E**) Effect of RhebL1 overexpression on AKT activation. (**F**) Effects of of G protein function of RhebL1 on AKT activation. In (B), A549 cells were stained with the indicated antibodies. Nuclei were stained with DAPI (blue). Scale bars, 10 μm. In (D), A549 cells were transfected with RhebL1 siRNA (50 nM) or control siRNA (50 nM) and subsequently stimulated with (or without) SPC (5 μM) for 1 h. In (E), for gene overexpression of RhebL1, A549 cells were transfected with a plasmid containing RhebL1 or an empty vector control (4 μg) and then treated with (or without) SPC (5 μM) for 1 h. Cell lysates were analyzed by Western blotting. In (F), A549 cells were transfected with a plasmid containing RhebL1Q64L or RhebL1D60K (4 μg) and then treated with (or without) SPC (5 μM) for 1 h.

To confirm binding, co-IP was performed using AKT1 antibody, and RhebL1 antibody. Co-IP of RhebL1 with AKT1 was carried out when cells were untreated or treated with SPC (Figure 4A). The binding of RhebL1 to AKT1 was confirmed by confocal microscopy (Figure 4B). Subsequently, we examined the effects of the G protein activity of RhebL1 on its binding to AKT1 using RhebL1Q64L (constitutively active) and RhebL1D60K (dominant negative). The binding of RhebL1 to AKT1 is dependent on its G protein activity (Figure 4C). We observed that endogenous RhebL1 was co-precipitated with AKT1 even in RhebL1D60K expressed cells (Figure 4C). This strongly supports that dominant mutant RhebL1D60K could not bind to AKT1.

We then examined the involvement of RhebL1 expression in SPC-induced AKT1 phosphorylation. SPC induced the phosphorylation of T308 and S473 in AKT1 and this effect was inhibited by gene silencing of RhebL1 in A549 cells (Figure 4D). In contrast, overexpression of RhebL1 induced the phosphorylation of T308 and S473 in AKT1 even without SPC treatment in A549 cells (Figure 4E). Moreover, we examined the effects of the G protein activity of RhebL1 on AKT1 phosphorylation of T308 and S473. RhebL1 and RhebL1Q64L (constitutively active) induced phosphorylation of AKT1 without SPC treatment, however, RhebL1D60K (dominant negative) did not (Figure 4F). These results suggest that G protein activity of RhebL1 is important in the binding and activation of AKT1.

### AKT1 is involved in the SPC-induced phosphorylation and reorganization of K8

**Figure 5:**
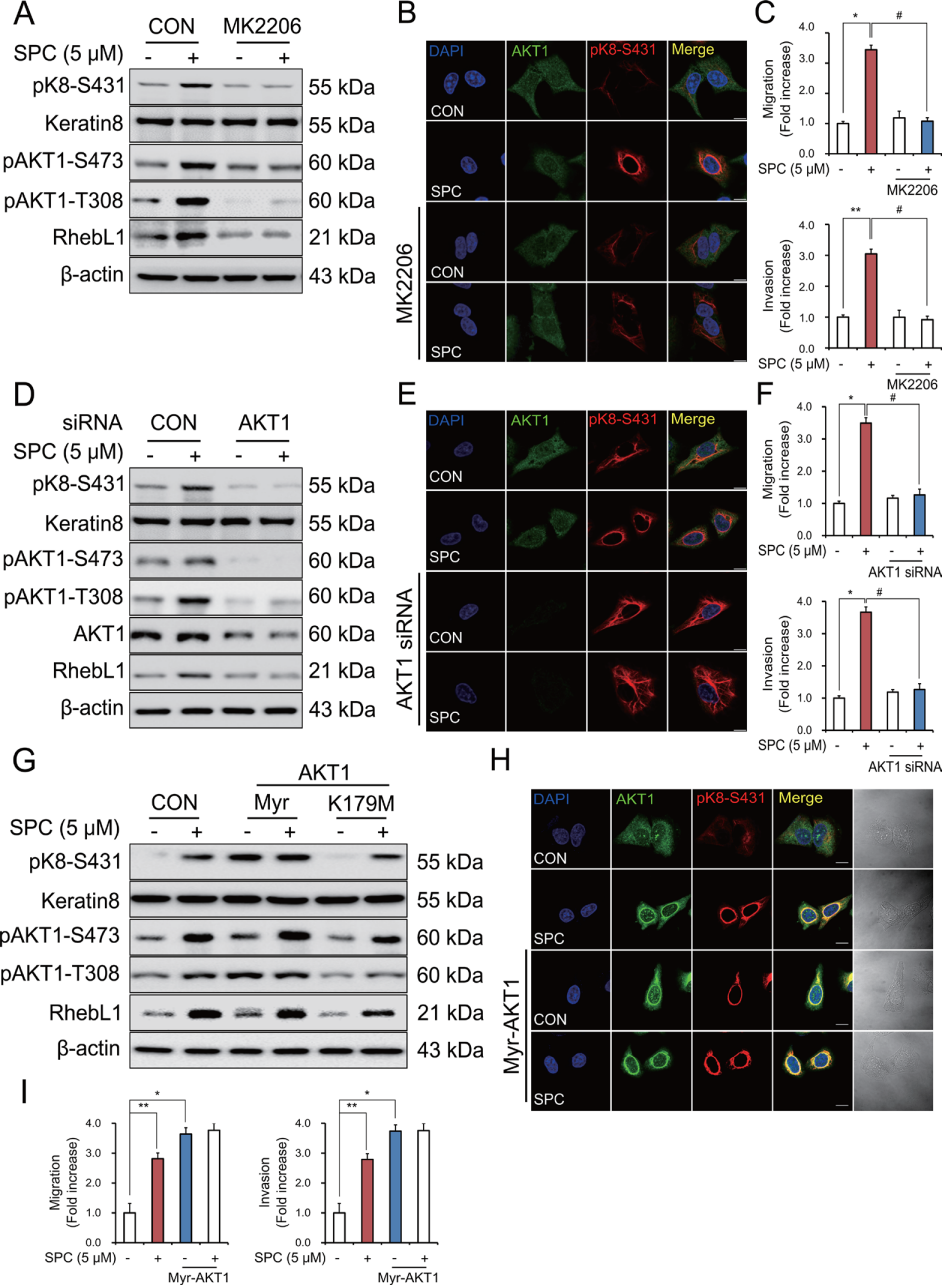
AKT1 is involved in the SPC-induced RhebL1 expression in A549 cells (**A**) Effect of AKT inhibitor, MK2206 on SPC-induced RhebL1 expression and K8 phosphorylation in A549 cells. A549 cells were treated with MK2206 (50 nM) for 1 h prior to SPC (5 μM) treatment. (**B**) Effect of MK2206 on SPC-induced perinuclear keratin organization in A549 cells. Scale bars, 10 μm. (**C**) Effects of MK2206 on SPC-induced migration and invasion of A549 cells. (**D**) Effect of AKT1 siRNA on SPC-induced K8 phosphorylation. Cell lysates were analyzed by Western blotting. (**E**) Effect of AKT1 siRNA on SPC-induced perinuclear reorganization of K8. Scale bars, 10 μm. (**F**) Effects of AKT1 siRNA on SPC-induced migration and invasion. (**G**) Effect of Myr-AKT1 overexpression on SPC-induced K8 phosphorylation. (**H**) Effect of Myr-AKT1 overexpression on SPC-induced perinuclear reorganization of K8. Scale bars, 10 μm. (**I**) Effects of Myr-AKT1 overexpression on SPC-induced migration and invasion. In (A–C), A549 cells were treated with or without 5 μM SPC for 1 h in the presence of MK2206 (AKT inhibitor, 50 nM). In (D–F), for gene silencing of AKT1, A549 cells were transfected with AKT1 siRNA (50 nM) or control siRNA (50 nM) and subsequently stimulated with (or without) SPC (5 μM) for 1 h. In (G–I), for overexpression of Myr-AKT1, A549 cells were transfected with a plasmid containing Myr-AKT1 or an empty vector control (4 μg) and then treated with (or without) SPC (5 μM) for 1 h. In (C), (F), and (I), cells were subsequently counted under four randomly chosen high-power fields (20×). The results are representative of 3 independent experiments with similar results (*n* = 3). *P < 0.05, **P < 0.01 compared with the control group. ^#^*P* < 0.05, ^##^*P* < 0.05 compared with the SPC-treated group.

To determine whether AKT1 was directly involved in the SPC-induced phosphorylation and reorganization of K8, we examined the effects of AKT1 inhibitor, MK2206 on SPC-induced events. MK2206 markedly suppressed SPC-induced phosphorylation and reorganization of K8 (Figure 5A, 5B), migration and invasion of A549 cells (Figure 5C).

Accordingly, gene silencing of AKT1 inhibited SPC-induced K8 phosphorylation and reorganization (Figure 5D, 5E) and migration and invasion of A549 cells (Figure 5F). Next, we examined whether kinase activity of AKT1 was involved in the SPC-induced events. Surprisingly, overexpression of myristoylated, active form of AKT1 induced K8 phosphorylation, whereas K179M, dominant negative form of AKT1 did not (Figure 5G). We also examined whether active form of AKT1 induced reorganization of K8, migration and invasion of A549 cells. Active form of AKT1 induced reorganization of K8, migration and invasion of A549 cells even without SPC treatment (Figure 5H, 5I). Together, these results suggest that AKT1 is involved in the SPC-induced K8 phosphorylation and reorganization, migration, and invasion of A549 cells.

### Effects of expression RhebL1 or RhebL1 & AKT1 on the prognosis of lung cancer patients

**Figure 6:**
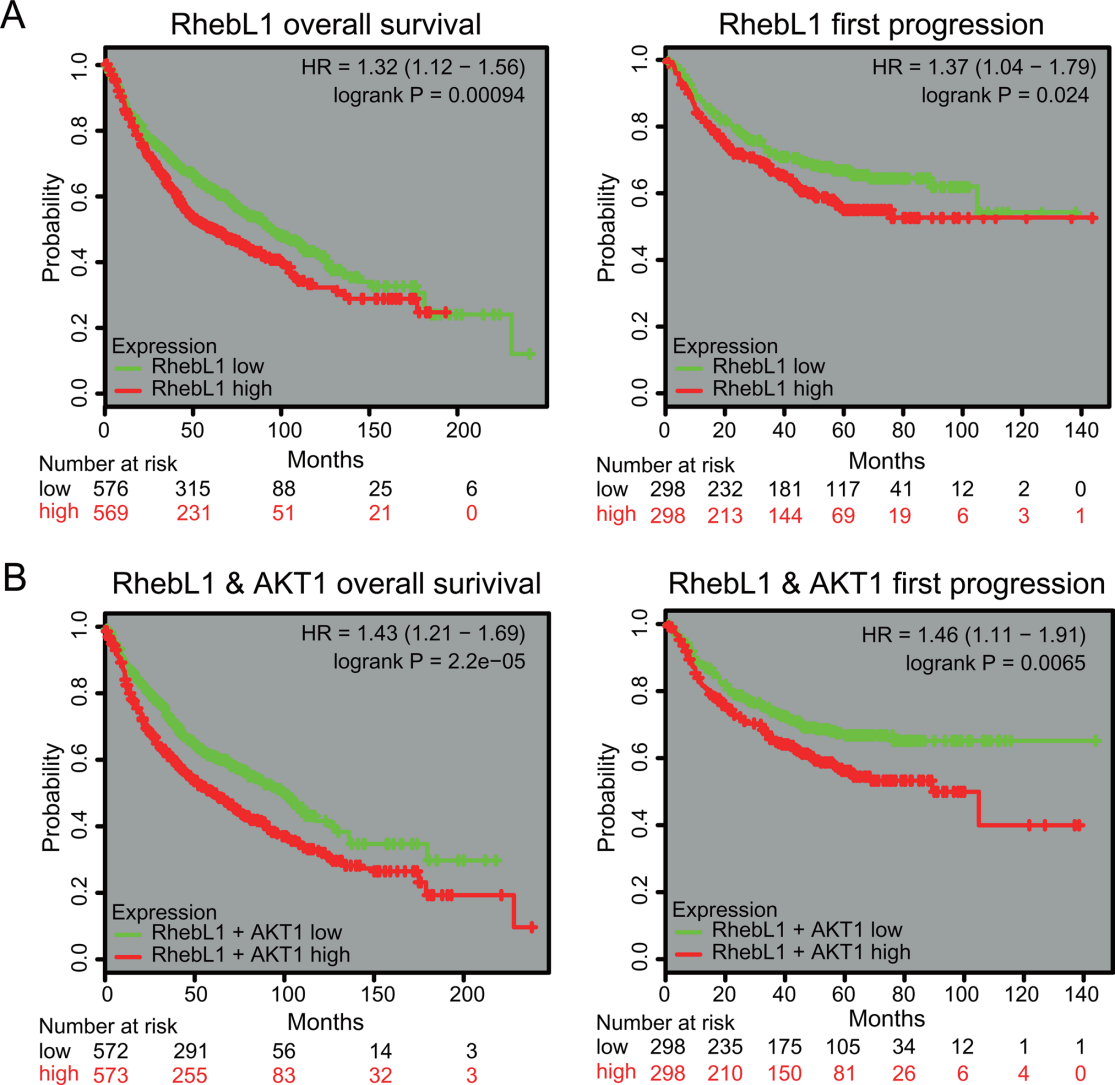
Effects of expression RhebL1 or RhebL1 & AKT1 on the prognosis of lung cancer patients (**A**) Effects of RhebL1 expression on survival of lung cancer patients. Overall survival curves were shown between RhebL1-high and RhebL1-low in lung cancer patients (left panel, *n* = 1145). Progression free survival curves between RhebL1-high & AKT1-high and RhebL1-low & AKT1-low in lung cancer patients (right panel, *n* = 596) are shown. (**B**) Effects of RhebL1 and AKT1 expression on survival of patients with lung cancer. Overall survival curves between RhebL1-high & AKT1-high and RhebL1-low & AKT1-low in lung cancer patients (*n* = 1145) are shown in the left panel. Progression free survival curves between RhebL1-high & AKT1-high and RhebL1-low & AKT1-low in lung cancer patients (*n* = 596) are shown in the right panel.

To investigate the prognostic significance of RhebL1 mRNA expression in lung cancer, survival analysis was done using online Kaplan Meier-plotter [35]. Lung cancer patients were divided into two subgroups (RhebL1-high and RhebL1-low) based on their median expression level. We found that the overall survival was high in lung cancer patients having low expression level of RhebL1 (*n* = 1145, *p* = 0.00094). Progression free survival was short in patients having high expression level of RhebL1 (*n* = 596, *p* = 0.024) (Figure 6A). In addition, we compared ‘RhebL1-high & AKT1-high’ with ‘RhebL1-low & AKT1-low’ using KMPLOT. Lung cancer patients were divided into two subgroups (‘RhebL1-high & AKT1-high’ and ‘RhebL1-low & AKT1-low’) on the basis of their median expression level. Survival curves showed a significant difference in the probability of overall survival (*n* = 1145, *p* = 0.000022, HR = 1.43) and progression free survival (*n* = 596, *p* = 0.0065, HR = 1.46) between the two groups (Figure 6B).

## DISCUSSION

We found that RhebL1 was increased in A549 or other lung cancer cells with SPC treatment (Figure 1A). Interestingly, the expression patterns of RhebL1 were similar to those of K8 in A549 cells (Figure 1D). Why does SPC promote expression of RhebL1? It is a difficult question to answer. It seems to be related to pleiotropic actions of SPC. The most plausible explanation might be based on the reports that SPC activates mTOR pathway and mTOR is required for endocytosis [36, 37]. Interestingly, SPC induces endocytosis via lipid raft [38, 39]. Therefore, SPC might induce the expression of RhebL1 to activate the mTOR pathway which is required for endocytosis.

Gene silencing of RhebL1 suppressed the SPC-induced K8 phosphorylation and reorganization (Figure 2A, 2B) and ectopic expression of RhebL1 induced SPC-induced K8 phosphorylation and reorganization (Figure 2D, 2E). Furthermore, gene silencing of RhebL1 inhibited the SPC-induced migration and invasion and overexpression of RhebL1 increased the migration and invasion, even without SPC treatment (Figure 2C, 2F).

We found that the G protein activity of RhebL1 is important in SPC-induced K8 phosphorylation and reorganization, migration and invasion (Figure 3). Overexpression of RhebL1D60K (dominant negative) with SPC treatment increased K8 phosphorylation at S431 or AKT1 phosphorylation (Figures 3A, 4F). This might be due to increased expression of endogenous RhebL1 by SPC. In fact, we confirmed SPC-induced endogenous RhebL1 expression by Western blot (Figures 3A, 4F). To the best of our knowledge, there have been no reports regarding the relationship between the G protein activity of RhebL1 and keratin- related events, although the dominant negative form of RhebL1 devoid of G protein activity is known to lead to a reduction in NF-κB activation [31]. Therefore, our results are the first report regarding the involvement of the G protein activity of RhebL1 in keratin reorganization.

What is the major effects of SPC on RhebL1? Is it up-regulation of gene expression of RhebL1 within 60 min or activation of RhebL1? We think that SPC-induced expression of RhebL1 is more important than the activation of RhebL1 since basal expression level of RhebL1 is lower than that of SPC-treated condition. In addition, GTP concentration might be enough for increased expression of RhebL1. RhebL1 GTPase is known to be a direct target of TSC2 GTPase activating protein (GAP) [33]. Moreover, AKT inhibits GAP activity of TSC2 for Rheb GTPase [40]. However, we do not know what is the effect of SPC on RhebL1 guanine nucleotide exchange factor (GEF) since we do not know which molecule acts as RhebL1GEF.

How is RhebL1 involved in keratin phosphorylation and reorganization? We speculate that RhebL1 might interact with molecules inducing kinase pathway, such as ERK, JNK and PKC leading to phosphorylation of K8 since we and other group have already reported that SPC-induced ERK, and JNK phosphorylate S431 of K8 [9, 41]. AKT1 might be a binding partner of RhebL1 in breast cancer cells [42]. We confirmed the binding of RhebL1 to AKT1 by co-immunoprecipitation (Figure 4A). G protein activity of RhebL1 increased the binding of it to AKT1 (Figure 4E). Overexpression of wild type RhebL1 induced phosphorylation of T308 and S473 in AKT1 even without SPC treatment (Figure 4F). SPC treatment did not induce further increase of phosphorylation of AKT1.

It is interesting that RhebL1 might bind to and activate AKT1 (Figure 4). A paralog of RhebL1, Rheb1, is inhibited by TSC2 and stimulates phosphorylation of S6K and 4EBP1 via mTORC1 activation [40]. AKT1 phosphorylates the TSC complex leading to relief of its inhibition of Rheb1 [43]. Our data suggests that RhebL1 may act as an AKT1 activator, however, it is not clear whether Rheb1 may also act as an AKT1 activator or whether the action of RhebL1 on AKT1 is unique.

MK2206, a well-known AKT inhibitor, suppressed the SPC-induced K8 phosphorylation and reorganization (Figure 5A, 5B). MK2206 is almost equally potent for human AKT1 and human AKT2 (IC_50_, 5 nmol/L and 12 nmol/L, respectively) and is about five-fold less potent against human AKT3 (IC_50_, 65 nmol/L) [44]. Therefore, we examined the involvement of AKT1 in SPC-induced K8 phosphorylation and reorganization using gene silencing and overexpression of AKT1. Inhibition of AKT or silencing AKT1 resulted in no induction of RhebL1 in the SPC treatment, leading to no induction of K8 phosphorylation (Figure 5A, 5D). Therefore, it seems that there are two roles of AKT1 in the SPC-induced actions. First, SPC-induced induction of RhebL1 expression needs AKT1 signaling. Second, SPC-induced RhebL1 binds to and activates AKT1. This AKT1 activation is important for the K8 phosphorylation, reorganization, migration of cancer cells. Interestingly, it is already known that overexpression of AKT1 increases K8/18 proteins [45]. Furthermore, AKT1 binds to K8, which probably contributes to the reciprocal hyperglycosylation and hypophosphorylation of AKT1 that occurs on K18 hypoglycosylation [46].

It is not clear how AKT1 induces the phosphorylation of S431 in K8. AKT1 phosphorylates protein phosphatase 1 (PP1) leading to inactivation of PP1 and this might result in ERK activation causing the phosphorylation and reorganization of K8 [47]. SPC decreased the expression of PP2A, and AKT1 is dephosphorylated by PP2A [39, 48]. SPC-induced RhebL1 activity may suppress the dephosphorylation of AKT1 by PP2A via the binding of RhebL1 to AKT1. To prove these speculations, further studies are undergoing.

In addition, it is currently unclear whether AKT1 is downstream or upstream of RhebL1 in SPC-induced K8 phosphorylation and reorganization. It is very difficult to define clearly the hierarchy between RhebL1 and AKT1 since mTOR is a downstream effector of Rheb1 or RhebL1 and mTOR phosphorylates S473 of AKT1, leading to fully active form of AKT1 [33, 49]. Our speculation about hierarchy based on canonical mTOR pathway is that SPC treatment induces endocytosis, leading to AKT1 activation which suppresses TSC1/TSC2 (RhebL1GAP activity) [50]. AKT1-induced suppression of RhebL1GAP increases RhebL1 activity which might activate mTOR [33]. However, AKT1 might be involved in RhebL1 expression (Figure 5). We do not know how AKT1 induces RhebL1. Furthermore, RhebL1 expression also induces AKT activation (Figure 4E, 4F).

We examined the effects of rapamycin on K8 phosphorylation and RhebL1 expression to determine the hierarchy among mTOR, RhebL1 expression, and RhebL1-induced K8 phosphorylation. mTOR inhibitor did not suppress RhebL1 expression or K8 phosphorylation (Supplementary Figure 2). These results suggest that canonical AKT/RhebL1/mTOR signaling did not fully explain the hierarchy between RhebL1 and AKT1 in aspects of K8 phosphorylation. We speculate that there might exist mTOR-independent novel mechanism among RhebL1, AKT1, and K8 phosphorylation. Studies about the novel mechanism among these players are undergoing.

The collective findings indicate that RhebL1 is involved in SPC-induced events in A549 lung cancer cells by activation of AKT1. These results suggest that suppression of RhebL1 or inhibition of binding of RhebL to AKT1 might be a novel means to prevent the malignant changes in the physical properties of metastatic cancer cells.

## MATERIALS AND METHODS

### Materials

RPMI 1640 and fetal bovine serum (FBS) were purchased from Welgene, Inc. (Daegu, South Korea). Lipofectamine™ 2000 Reagent was purchased from Invitrogen (Carlsbad, CA). D-erythro- and L-threo-SPC were purchased from Matreya (Pleasant Gap, PA). MK2206 was purchased from Santa Cruz Biotechnology (Santa Cruz, CA). Anti-AKT1 and phospo-specific antibodies to detect K8 S431 were supplied from Abcam (Cambridge, UK). Anti-RhebL1 antibody and peroxidase-labeled secondary antibodies were acquired from Santa Cruz Biotechnology. Anti-phospho-AKT Ser 473 was acquired from Cell Signaling Technology (Beverly, MA). Alexa Fluor 488 donkey anti-goat antibody and Alexa Fluor 594 chicken anti-rabbit antibody were obtained from Molecular Probes, Inc. (Eugene, OR).

### Cell culture

Human lung cancer cell lines, including A549, H1299, H1703 and H838 were obtained from the American Type Culture Collection (Rockville, MD). All cells were maintained in RPMI 1640 medium supplemented with 10% heat-inactivated fetal bovine serum FBS and penicillin-streptomycin (10,000 μg/ml). The cells were grown at 37°C in a humidified atmosphere containing 5% CO_2_.

### Western blot

The cell lines A549, H1299, H1703 and H838 were harvested and lysed in 50 mM Tris-Cl (pH 7.5), 150 mM NaCl, 1% Triton X-100, 1% sodium deoxycholate, 0.1% sodium dodecyl sulfate (SDS), 2 mM EDTA, and protease inhibitors (Gendepot, Barker, TX). The protein concentrations of the supernatants were determined using Coomassie Plus (Thermo Scientific Inc., Rockford, IL), as recommended by the manufacturer. Proteins in the lysates were separated by 10% SDS-polyacrylamide gel electrophoresis (SDS-PAGE) and transferred to polyvinylidene difluoride membranes (Pall, Pensacola, FL). Membranes were blocked with 3% non-fat milk and probed with the appropriate primary antibody, which included anti-K8 S431, anti-RhebL1, and anti-AKT1 Thr 308 and Ser473. After primary-antibody incubation, the membrane was washed with Tris buffered saline (TBS) including 0.1% Tween 20. The membrane was further incubated for 1 h with a peroxidase-conjugated secondary antibody (1:5000, Santa Cruz Biotechnology) at room temperature. Immunoactive proteins were detected using the Power Opti-ECL Western blotting detection reagent (Animal Genetics Inc., Gyeonggi, Korea).

### Reverse transcription-PCR (RT-PCR)

Total RNA was extracted from cells using the Tri-Reagent method (Invitrogen) according to the manufacturer's instructions in a RNase-free environment. Reverse transcription of 1 μg RNA was carried out using M-MuLV reverse transcriptase (Promega, Madison, WI), oligo (dT)15 primer, dNTP (0.5 μM), and 1 U RNase inhibitor. PCR was performed with the Applied Biosystems GeneAmp PCR system (Invitrogen); the amplification program included 30 cycles at 94°C for 30 s (denaturing), 55°C for 30 s (annealing) and 72°C for 30 s (extension). Subsequently the PCR products were electrophoresed on a 1% agarose gel.

### Transfection with small-interfering RNA (siRNA) or plasmid DNA

For transient knockdown of RhebL1 siRNA, RhebL1 siRNA (5′- GCA GGA UGA GUA CAG CAU U-3′) and universal negative siRNA as a negative control was purchased from Samchully Pharm. The RhebL1 open-reading frame (ORF)-containing plasmids pET28-MHL was purchased from Addgene (Cambridge, MA) and subcloned into pcDNA3.1-HA. RhebL1 D60K/Q64L plasmids were generated by site directed mutagenesis (Cosmogenetech, Seoul, Korea). The AKT1 ORF-containing plasmids pcDNA3.1 Myr-HA-AKT1-WT and K179M were kindly provided by Jae Whan Song (Yonsei University). For siRNA or plasmid DNA transfection experiments, the cells were plated on 6-well plates until they reached 50% confluence and were then transfected with RhebL1 siRNA (50 nM) or plasmid containing RhebL1 using Lipofectamine™ 2000 (Invitrogen) in Opti-MEM medium (Gibco, Grand Island, NY) according to the manufacturer's instruction. Six hours after transfection, A549 cells were grown in complete culture medium. After this, the cells were starved for 15 h and treated with SPC (5 μM) for 1 h. Proteins were extracted from the cells and analyzed by Western blot.

### Invasion and migration assay using Transwell plates

Migration assays were performed using a multi-well chamber (Neuroprobe Inc., Gaithersburg, MD) coated with 10 μg/ml fibronectin as a chemoattractant. Briefly, A549 cells were suspended in RPMI1640 at a density of 1 × 10^6^ cells/ml, and a 25 μl aliquot of this suspension was placed in the upper well of the chamber. Next, the aliquot was separated from 3% serum (in the lower well) by an 8 μm polyhydrocarbon filter. After 37^°^C incubation for 6 h, non-migrated cells on the upper surface of the membrane were scraped off, and the migrated cells on the lower surface were stained by Diff-quick, and subsequently counted in five randomly chosen high-power (200×) fields.

Invasion assays were performed using a 24-well Transwell unit with polycarbonate filters (diameter: 6.5 mm, pore size: 8.0 mm; Costar Corning, Corning, NY). Trypsinized cells were suspended in serum-free medium, and 2 × 10^5^ cells were added to the upper chamber of the Transwell inserts. After 15 h cell incubation, the non-migrated cells on the upper surface of the membrane were scraped off, and those on the lower surface were stained using the hematoyxylin and eosin (H&E) staining system (Fisher Scientific, Houston, TX), photographed at 200 × magnification, and counted in five randomly selected fields. All of the treatments were performed in triplicate wells.

### Immunofluorescence staining

Cells were seeded onto coverslips and transfected with RhebL1 siRNA or plasmid DNA containing RhebL1. The cells were treated with SPC (5 μM) for 1 h. Cells were fixed with 4% paraformaldehyde (PFA) for 10 min at room temperature and permeabilized with 0.5% Triton X-100 for 15 min followed by several washes with phosphate buffered saline (PBS). After blocking with 3% bovine serum albumin (BSA) in PBS at room temperature for 1 h, coverslips were incubated with anti-K8 S431, anti-RhebL1, anti-AKT1, and anti-AKT1 Thr 308 and Ser473 primary antibodies overnight at 4°C. Excess antibody was removed with PBS and then species-specific secondary antibodies conjugated to chicken anti-rabbit IgG antibody (Alexa Fluor 594, 1:500 Molecular probes) and donkey anti-goat IgG antibody (Alexa Fluor 488, 1:500 Molecular probes) were reacted with the coverslips for 1 h at room temperature. After four washes in PBS, the slides were mounted with mounting solution and visualized at 100 × magnification using a Nikon confocal microscope.

### Immunoprecipitation (IP)

The cells were lysed in IP lysis/wash buffer, and 1 mg of lysate was incubated with anti-AKT1 (rabbit polyclonal), anti-RhebL1 (goat polyclonal), anti-HA (goat polyclonal) and anti-K8 S431 (rabbit polyclonal) antibodies overnight at 4°C. Protein A/G magnetic beads (Pierce, Rockville, IL) were added (25 μL each) to each sample tube and incubated for 1 h. Eluted SDS sample volumes of 15 μL were resolved by SDS-PAGE and analyzed by Western blot.

### Statistical analysis

The student's *t*-test was used to determine the statistical significance of the differences between the experimental and control group values. Data are expressed as means ± standard of error measurement (S.E.M.) of at least 3 independent experiments performed in triplicate. *P* < 0.05 was considered statistically significant.

## SUPPLEMENTARY MATERIALS FIGURES


