# Supplementary material for: Novel involvement of RhebL1 in sphingosylphosphorylcholine-induced keratin phosphorylation and reorganization: Binding to and activation of AKT1
Source: Oncotarget. 2017 Feb 15;8(13):20851–64. doi: 10.18632/oncotarget.15364 (PMC5400551; doi:10.18632/oncotarget.15364)
Supplement: Supplementary file 1 [file oncotarget-08-20851-s001.pdf]

Novel involvement of RhebL1 in sphingosylphosphorylcholine-induced keratin phosphorylation and reorganization: binding and activation of AKT1

Supplementary Materials

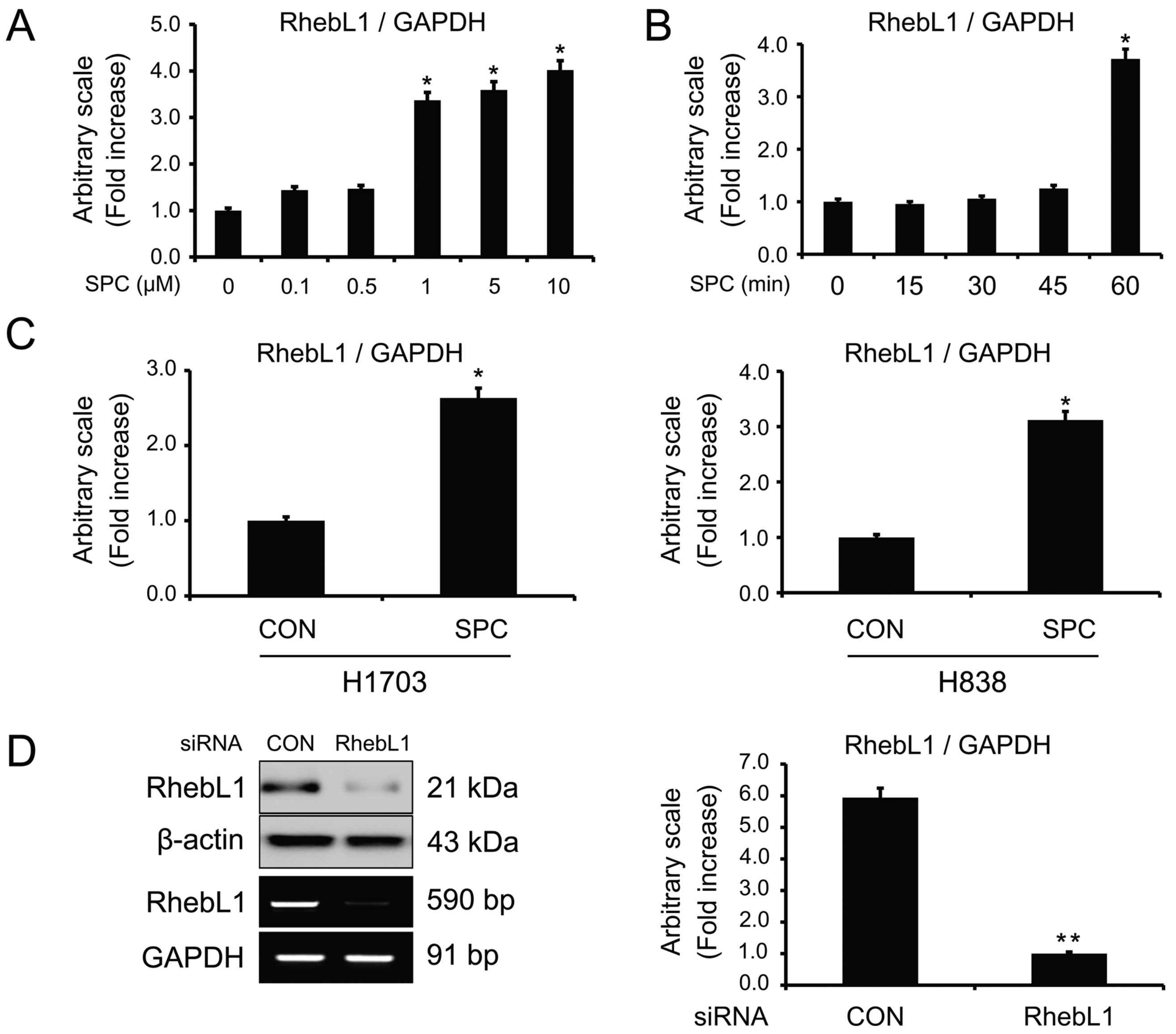

Supplementary Figure 1: (**A**) Intensities of bands on RT-PCR shown in Figure 1A were quantified by densitometric analysis. (**B**) Intensities of bands on RT-PCR shown in Figure 1B were quantified by densitometric analysis. (**C**) Intensities of bands on RT-PCR shown in Figure 1C were quantified by densitometric analysis. (**D**) Effects of RhebL1 siRNA in A549 cells. Cell lysates were analyzed by Western blotting and RT-PCR. Intensities of bands on RT-PCR shown in D were quantified by densitometric analysis. *$P < 0.05$, **$P < 0.01$ compared with the control group.

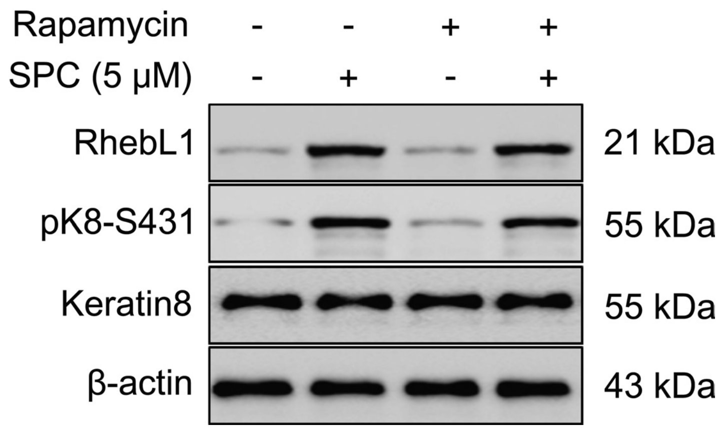

Supplementary Figure 2: Effect of Rapamycin on SPC-induced RhebL1, K8 phosphorylation and AKT1 phosphorylation in A549 cells. A549 cells were treated with Rapamycin (100 nM) for 1 h prior to SPC (5 μM) treatment. Cell lysates were analyzed by Western blotting.
